# House Building

**Published:** 1879-03

**Authors:** 


Wooden houses are warm and dry, and for the country as well as for town and country in the south, are greatly to be preferred. Damp dwellings originate consumption in its most insidious and resistless forms.

If a house is built of brick or stone, the plastering should never be laid on the wall itself; the wall should be lathed, so as to have an inch or more between the brick or stone and the lathing, on which the plaster should be spread.

When in Havana de Cuba some years ago, for information, not health, I observed that the island was composed of limestone, so soft that a common pick-axe would dig out the cellar in large blocks of stone, out of which the building itself was erected; and not only were these large stones used, but the smaller ones, not an inch in diameter, by using mortar largely between the large stones, while the small ones were stuck into the mortar; in some cases the small stones and mortar seemed to predominate; but from the hour of building, the wall became harder and harder. The large stones themselves increased in hardness, so that in the course of a few years the wall becomes almost one solid piece of marble, from the influence of moisture and carbonic acid absorbed by the soft lime. On the same principles, great nature made the conglomerate variegated marble columns in the capital at Washington.

It is by copying after nature, man makes his greatest and most useful discoveries. But to get at nature's processes, discover the secret of her operations, requires often long years of anxious study, of perplexing conjecture, of ruinous expenditure, of wasting discouragement; not unfrequently the brain itself gives way, and the noble spirit is a wreck and ruin, and goes forgotten to an unhonored grave. Sooner or later some other man, more fortunate, takes up the investigation where he left it off, and by being able to bring a store of unimpaired energies to the work, pursues it to a successful issue. We glorify the commonest soldier who has perished on the battle-field in trying to kill his brother man, but these unsuccessful strivers after great practical truths, our hardy mechanics whose hands are scarred and seared with labor, whose joints have stiffened and whose bodies have grown bent by incessant stooping toil, these are perishing every day, without a drum or funeral note, or word of pity or of praise, except indeed to the successful few.

